# Uninvestigated dyspepsia and associated factors of patients with gastrointestinal disorders in Dessie Referral Hospital, Northeast Ethiopia

**DOI:** 10.1186/s12876-017-0723-5

**Published:** 2018-01-18

**Authors:** Abdurahaman Seid, Zemenu Tamir, Wondmagegn Demsiss

**Affiliations:** 1 Department of Medical Laboratory Sciences, College of Medicine and Health Sciences, Wollo University, Po. Box 1145, Dessie, Ethiopia; 2 Department of Medical Laboratory Sciences, College of Health Sciences, School of Allied Health Sciences, Addis Ababa University, Addis Ababa, Ethiopia; 3 Department of Medical Laboratory Sciences, College of Medicine and Health Sciences, Wollo University, Dessie, Ethiopia

**Keywords:** *H. pylori*, Uninvestigated dyspepsia, Rome III, Self-reported, Ethiopia

## Abstract

**Background:**

Dyspepsia is a common problem in the community and clinical practice with symptom(s) considered arising from the gastroduodenal region. Dyspepsia burden and associated factors vary from country to country. The aim of this study was to determine the prevalence of uninvestigated dyspepsia (UD) using Rome III criteria, associated risk factors and self-reported dyspepsia symptoms’ correlation with *H. pylori* infection.

**Methods:**

A cross-sectional study was conducted among randomly selected 318 out patients with gastrointestinal complaints during the period from September 1 to December 30, 2015. All patients completed a questionnaire for collecting data regarding sociodemographic, lifestyle and functional gastrointestinal disorders. Diagnosis of dyspepsia was made according to the Rome III criteria. *H. pylori* infection was assessed using stool antigen test. SPSS version 20.0 statistical software package was used for data analysis.

**Results:**

From a total of 318 patients, 48.4% had UD according to Rome III criteria; with 42.1% symptoms of epigastric pain/burning, 26.1% postprandial fullness and 22.6% early satiation. Epigastric pain/burning (AOR = 1.92, 95% CI 1.07–3.43), early satiation (AOR = 2.68, 95% CI 1.38–5.20) and belching (AOR = 4.7, 95% CI 1.54–14.40) were significantly correlated with *H. pylori* infection. *H. pylori* infection (AOR = 4.33, 95% CI 2.41–7.76) and aspirin/NSAIDs consumption (AOR = 5.29, 95% CI 2.82–9.93) were independent risk factors for UD. However, consumption of raw fruits/ vegetables at least once a week (AOR = 0.48, 95% CI 0.24–0.98) and taking two or more cups of tea a day (AOR = 0.339, 95% CI 0.17–0.70) were inversely associated with UD.

**Conclusions:**

UD is highly prevalent among adults with gastrointestinal complaints. *H. pylori* infection is significantly associated with UD and correlates with its symptoms. Individuals with epigastric pain/burning, early satiation and belching should be primary focus of *H. pylori* infection diagnosis and treatment. The role of consumption of tea, raw fruits and vegetables on dyspepsia needs further large scale study.

## Background

Functional gastrointestinal disorders (FGIDs) are highly prevalent in the general population and are frequent cause for consultation of a general practitioner [1]. Individuals with FGIDs contribute heavily to healthcare utilization [2]. The Rome III criteria for diagnosis of FGIDs were developed in 2006 [3], and since then a few studies have estimated prevalence of FGIDs, described associated factors, signs and symptoms using this criteria [4].

Dyspepsia is one of a common symptom complex of FGIDs with an extensive differential diagnosis and a heterogeneous pathophysiology. According to Rome III definition, dyspepsia is “chronic or recurrent pain and/or discomfort thought to originate from the gastroduodenal region” and at least having one or more of the following symptoms: postprandial fullness (PPF), early satiation (ES), epigastric pain or burning [5–7]. Moreover, one symptom must be present for at least the last 3 months with an onset of symptoms at least 6 months prior to diagnosis [7].

Patients with symptoms of dyspepsia but didn’t undergo any investigations such as upper gastrointestinal (GI) endoscopy and is not categorized as functional or organic in etiology are classified as having uninvestigated dyspepsia (UD), whereas those who undergo investigation and have no detectable cause for their symptoms are considered to have functional dyspepsia (FD) [6]. Numerous cross-sectional surveys reported different prevalence of dyspepsia in the community which varies from 7% to 54.6%, and globally estimated that 25% of the general populations suffer from dyspeptic symptoms [8–12]. High prevalence of UD (41.2%) was observed in the general population with 70–80% of patients with dyspepsia had no clinically significant findings at endoscopy [12]. Recently conducted systematic review and meta-analysis has demonstrated 21% pooled prevalence of UD, but varied among countries (1.8% to 57.0%) and according to the criteria used to define its presence [13].

Dyspepsia complaints are the most common reasons for outpatient visits to primary care providers [5, 14] and 20% to 40% gastroenterology appointments [14]. Both in an uninvestigated dyspeptic and FD patients, the most prevalent symptoms are PPF (68–86%), upper abdominal bloating (68–84%), epigastric pain (68–74%), ES (49–72%) [15, 16], belching (50–60%), epigastric burning (35–57%), nausea (39–65%) and vomiting (23–31%) [16].

The causes of dyspepsia remain uncertain as being females and underlying psychological disturbances contribute to FD, whilst environmental/lifestyle factors such as poor socio-economic status, smoking, increased caffeine intake and ingestion of non-steroidal anti-inflammatory drugs (NSAIDs) appear to be more related to UD [17]. Moreover, the role of *H .pylori* infection in dyspepsia is still unclear. Some authors indicated no association between *H. pylori* infection and dyspepsia [18–21], whereas others indicated that dyspepsia may be a sign of acute or chronic *H. pylori* infection as evidenced from population-based study conducted in UK [22].

In Ethiopia, many hospital-based studies reported different prevalence rates of *H. pylori* infection among dyspeptic patients [21, 23–26], but have not estimated the burden of dyspepsia. Moreover, Rome III criteria have not been widely applied for diagnosis and classification of FGIDs. Thus, the proportion of dyspeptic patients and their frequent typical symptoms of individuals presented with FGIDs were not documented according to Rome III criteria. Furthermore, no study has been conducted concerning potential risk factors of dyspepsia.

The determination of dyspepsia burden along with its associated symptoms and potential risk factors is important to increase physicians’ ability of symptoms-based approach of sufferers to provide more precise diagnosis, treatment and prevention of the disorder particularly in resource limited settings like Ethiopia. Therefore, the aim of this study is to assess the prevalence of UD using Rome III criteria and its major symptom types of adults with FGIDs complaining patients. Furthermore, the present study is designed to assess associated risk factors of UD and correlation of *H. pylori* infection with self-reported functional upper GI symptoms.

## Methods

### Study patients and setting

This cross-sectional study was conducted at Dessie Referral Hospital, Dessie town, Northeast Ethiopia, from September 1 to December 30, 2015. The sample size was estimated to be 384 by taking 50% prevalence since there was no previous similar study in Ethiopia. The study population consisted of randomly selected new adult patients fulfilling the inclusion criteria at the outpatient department of the hospital.

We assessed the number of GI symptomatic patients who visited the hospital of previous comparative months of the study period. An average of 15 new GI symptomatic patients attended the outpatient department each day. In our country, working day of the month are Monday to Friday (5 days) giving a total of 22 days per month. The total expected new symptomatic patients attending the hospital during the study period were estimated to be 1320. The average number of study participants included per day, obtained by dividing the sample size to total working day of study period, was approximately 5. When the total population was divided by the sample size, the sample interval was found 3. The first person was selected by lottery method and then systematic random sampling method was used for selecting the patients at an interval of every 3 patients using the sequential order of their card number as a sampling frame.

### Inclusion criteria

The presence of at least one of the following GI complaints existing for at least 3 months with the onset of symptom at least 6 months before clinical visit. These includes abdominal pain, bloating, nausea, vomiting, ES, PPF, heartburn, regurgitation, epigastric pain/burning and belching.

### Exclusion criteria

GI symptomatic patients less than 18 years of age, and who took treatment against *H. pylori* infection within 3 months prior to this study, those who used proton pump inhibitors or H2-blockers for more than two weeks before study enrollment, presence of confirmed systemic disease such as congestive heart failure, coronary heart disease, liver failure, diabetes mellitus, thyroid disease, hematological diseases (for all case it is obtained by reviewing their medical documents), presence of major psychiatric disorders and pregnant mothers were excluded. Patients who were unable to communicate, and at emergency were also excluded.

### Questionnaires

Study participants were asked to complete the dyspepsia questionnaire consisting FD modules of FGIDs and scored using Rome III algorithm [27]. Sociodemographic data (age, gender, schooling level, residence, occupational and marital status) and life style (consumption of tea, coffee, spice food, raw fruits and vegetables, alcohol, and NSAIDs) related factors were also collected. We adapted a previously validated FD module of FGIDs questionnaire (English version) [27] and translated in to the local language, “Amharic” version, through a process of translation and back translation before data collection. “Amharic” is mother tongue of population in the study area.

The translation process was as follows: Two medical professional who were native speakers of” Amharic” and fluent in English, undertook translations independently. One professional translated the English version to “Amharic” and the other translated the “Amharic” version back to English. The original and back-translated (English) versions were compared to detect any misunderstandings, mistranslations, or inaccuracies in the “Amharic” draft questionnaire. Feed backs about the “Amharic” version of the FD module of FGIDs questionnaire was obtained from experts in gastroenterology. The translated questionnaire was administered to 19 participants with FGIDs to assess the clarity, understandability, and appropriateness of the translation’s wording so that the final version which contains 30 questions was used to collect required information. Similar procedure was followed for preparing “Amharic” version of the socio-demographic and life style related questionnaire. For participants who can’t read and write, interview was employed. The clinical residents explained the question and queries regarding the meaning of medical terms and elaboration of symptoms when asked by the patients.

Dyspepsia was assessed according to the Rome III criteria as having one or more of the following: PPF, ES, epigastric pain, and/or epigastric burning for the past three months with onset at least six months before diagnosis [3]. However, UD was not directly addressed by the Rome III criteria. We depended on the symptoms that were defined for FD by the Rome III criteria in order to recognize those with dyspepsia and since no upper GI endoscopy was performed, patients who fulfill the symptomatic criteria were diagnosed as UD. Therefore, individuals were classified as having UD if they have reported experiencing at least one of the following symptoms: (1) bothersome postprandial fullness happening after ordinary sized meals; (2) early satiation that prevents finishing a regular meal; (3) epigastric pain/burning localized to the epigastrium.

### Evaluation of *H. pylori* status and ABO blood group

About 20 g of fresh stool was collected from each participant in sterile and screw caped containers. The presence of *H. pylori* antigen was determined using monoclonal anti-*H.pylori* antibody conjugated with colloid gold nitrocellulose membrane strip (Rapid stool antigen test) based on a lateral flow chromatographic immunoassay technique. Specimens were tested using one step stool antigen test (Zhejiang Orient Gene Biotech CO., LTD, China) with 94.9%–100% sensitivity and 95–100% specificity [28]. Test results were read and interpreted according to the manufacturer’s instructions. This test kit is validated and approved by the ministry of health of the country and currently employed for the diagnosis of *H.pylori* infection in the country. Five ml of venous blood was collected and ABO blood grouping was performed by slide agglutination test using monoclonal anti-A, anti-B, anti-AB and anti-D (Rho) antibodies.

### Statistical analysis

All completed questionnaires were coded. Data analysis was performed using SPSS version 20 in four major steps. First, descriptive statistics were used to summarize the participant characteristics. The chi square (χ2) test was used to test the association between socio-demographic and lifestyle factors and dyspepsia. Possible risk factors for dyspepsia were initially examined using univariate analyses. Risk factors with *p* < 0.25 were entered to logistic regression model using stepwise backward logistic regression analysis to identify the risk factors independently associated with dyspepsia. All tests were two-tailed and the level of statistical significance was set at *p* < 0.05 along with the corresponding odds ratio (OR) and 95% confidence intervals (CI) in cases of significant associations.

### Ethical consideration

The study was commenced after ethically approved by the research ethics committee of Wollo University. Permission to conduct the study was also obtained from the hospital administration. Written informed consent was obtained from each study participant and the results were kept confidential. Any result that was necessary for the patient was communicated with the physician for appropriate management.

## Results

### Sociodemographic and life style characteristics

Among 383 randomly selected participants with GI complaints, 24 were not eligible (10 were below 18 years old, 14 were with symptom onset less than 6 months), and 41 returned incomplete questionnaire. Therefore, a total of 318 participants with complete information were included in the final analysis.

The mean (± SD) age of participants was 37.84 ± 12.41 years ranged from 18 to 77 years old, and 54.4% were females. Out of 318 participants, 46.9% were in the age group 30 to 49 years old, 67.4% were married and 61% were urban dwellers. Almost half of study participants had a habit of drinking alcohol, and 31.1% were actively infected with *H. pylori*. Sociodemographic and life style factors of study participants are described in Table 1.

**Table 1:** Socio-demographic and lifestyle characteristics of functional gastrointestinal symptomatic adult patients in Dessie Referral Hospital, Northeastern Ethiopia, 2015 (*n* = 318)

Variable		Freq	%	Variables		Freq	%
Age (year) mean ± SD	37.84 ± 12.41			Eating spicy food	Yes	269	84.6
					No	49	15.4
Age group	< 30 years	105	33.01				
	30 to 49 years	149	46.9	Tea consumption	Never	106	33.3
	≥50 years	64	20.12		One cup/day	96	30.2
Sex	Male	145	45.6		>one cup/day	116	36.5
	Female	173	54.4	Coffee consumption	Never	114	35.8
Marital status	Married	214	67.4		One cup/day	53	16.7
	Single	63	19.8		>one cup/day	151	47.5
	Divorced/widowed	41	12.9	Raw fruits and/or Vegetable consumption	Never	78	24.5
Residence	Rural	73	23		at least once/day	53	16.7
	Urban	194	61		at least once/week	187	58.8
	Suburban	51	16	Alcohol drinking	Yes	156	49.1
Occupation	Gov,t	108	34		No	162	50.9
	Student	29	9.1	Recent drug consumption	Never	187	58.8
	Farmer	66	20.8		Aspirin/NSAIDs	87	27.4
	Merchant	51	16.0		Other drugs	44	13.8
	Housewife	51	16.0	Blood group	Group A	82	25.8
	Others	13	4.1		Group B	89	28.0
Education	No legal education	50	15.7		Group AB	21	6.6
	Grade 1–8	74	23.3		Group O	126	39.6
	Grade 9–12	72	22.6	*H. pylori* infection	Yes	99	31.1
	College and above	122	38.4		No	219	68.9

### Dyspepsia and its associated factors

Of 318 participants with complaints of GI symptoms, according to Rome III criteria, 63.5% had at least one self-reported functional GI symptom with the prevalence of UD being 48.4%. Commonly reported upper GI symptoms were epigastric pain or burning, PPF, complain of ES and belching (Table 2).

**Table 2:** Frequency and distribution of self-reported functional gastrointestinal symptoms in study population using Rome III criteria, Dessie Referral Hospital, Northeastern Ethiopia, 2015(n = 318)

Functional gastrointestinal symptoms					Functional gastrointestinal symptoms			
			Freq	%			Freq	%
At least one symptom		Yes	202	63.5	UD & HB	Yes	78	24.5
		No	116	36.5		No	240	75.5
UD		Yes	154	48.4	Belching	Yes	80	25.2
		No	164	51.6		No	238	74.8
UD typical symptoms	PPF	Yes	83	26.1	vomiting	Yes	18	5.7
		No	235	73.9		No	300	94.3
	ES	Yes	72	22.6	Nausea	Yes	11	3.5
		No	246	77.4		No	307	96.5
	Epigastric burning/pain	Yes	134	42.1	UD & belching	Yes	66	20.8
		No	184	57.9		No	252	79.2
	PPF and ES	Yes	62	19.5	UD, HB & belching	Yes	43	13.5
		No	256	80.5		No	275	86.5

Key: UD = uninvestigated dyspepsia, PPF = postprandial fullness, ES = early satiation, HB = heart burn

Chi-square test showed that *H. pylori* infection was significantly associated with UD (χ2 = 21.33, *p* < 0.001) as were habit of taking aspirin and/or NSAIDs (χ2 = 30.27, p < 0.001), consumption of tea (χ2 = 18.23, p < 0.001) and raw fruit and/or vegetables (χ2 = 8.71, *p* = 0.01) (Table 4). However, no significant association observed between sex, age, residence, educational level, occupational and marital status (Table 3), ABO blood group, consumption of coffee and spicy foods, drinking of alcohol and UD (Table 4).

**Table 3:** The prevalence of uninvestigated dyspepsia in relation to demographic variables among study participants in Dessie Referral Hospital, Northeastern Ethiopia, 2015 (n = 318)

Variable		Total (N)	UD n (%)	χ 2	*p*-value
Sex	Male	145	67(46.2)	0.53	0.468
	Female	173	87(50.3)		
Age	< 30 years	105	47(44.8)	0.96	0.618
	30 to 49 years	149	76(51.0)		
	≥ 50 years	64	31(48.4)		
Marital status	Married	214	99(46.3)	3.00	0.223
	Single	63	30(47.6)		
	Divorced/widowed	41	25(61.0)		
Residence	Rural	73	38(52.1)	2.83	0.242
	Urban	194	87(44.8)		
	Suburban	51	29(56.9)		
Occupation	Gov,t	108	48(44.4)	9.96	0.076
	Student	29	16(55.2)		
	Farmer	66	38(57.6)		
	Merchant	51	20(39.2)		
	Housewife	51	29(56.9)		
	Others	13	3(23.1)		
Education	No legal education	50	25(50.0)	0.62	0.893
	Grade 1–8	74	38(51.4)		
	Grade 9–12	72	35(48.6)		
	College and above	122	56(45.9)

**Table 4:** The prevalence of uninvestigated dyspepsia in relation to lifestyle and other factors of study participants in Dessie Referral Hospital, Northeastern Ethiopia, 2015 (n = 318)

Variables		Total	UD = n/%	χ 2	P-value
Eating spicy food	Yes	269	132(49.1)	0.29	0.591
	No	49	22(44.9)		
Tea consumption	Never	106	59(55.7)	18.23	<0.000
	One cup/day	96	57(59.4)		
	>one cup/day	116	38(32.8)		
Coffee consumption	Never	114	54(47.4)	0.19	0.911
	One cup/day	53	27(50.9)		
	>one cup/day	151	73(48.3)		
Raw fruits and/or Vegetables consumption	Never	78	49(62.8)	8.71	0.013
	Eat at least once/day	53	22(41.5)		
	Eat at least once/week	187	83(44.4)		
Alcohol drinking	Yes	156	79(50.6)	0.60	0.438
	No	162	75(46.3)		
Recent drug consumption	Never	187	67(35.8)	30.27	<0.000
	Aspirin/NSAIDs	87	61(70.1)		
	Other drugs	44	26(59.1)		
Blood group	Group A	82	37(45.1)	6.51	0.089
	Group B	89	35(39.3)		
	Group AB	21	12(57.1)		
	Group O	126	70(55.6)		
*H. pylori* infection	Yes	99	67(67.7)	21.33	<0.000
	No	219	87(39.7)		

Exploring independent risk factors in a logistic regression model showed that *H. pylori* infection [AOR = 4.33, 95% CI 2.41–7.76, *p* < 0.001] and consumption of aspirin and/or NSAIDs [AOR = 5.29, 95% CI 2.82–9.93, p < 0.001] were independent predictors of UD. However, consumption of raw fruit and/or vegetables at least once a week [AOR = 0.48, 95% CI 0.24–0.98, *p* = 0.042] and tea consumption at least two cups a day [AOR = 0.34, 95% CI 0.17–0.70, *p* = 0.003] showed a protective effect against UD. No association was distinguished between consumption of one cup of tea a day, marital status, ABO blood group, type of occupation and UD (Table 5).

**Table 5:** Regression analysis of different factors associated with uninvestigated dyspepsia of study participants in Dessie Referral Hospital, Northeastern Ethiopia, 2015 (n = 318)

Variable		Total	UD = n(%)	COR(95%CI)	AOR(95%CI)	p-value
Marital status	Married	214	99(46.3)	ref		
	Single	63	30(47.6)	1.06(0.60--1.85)	–	–
	Divorced/widowed	41	25(61.0)	1.82(0.92--3.59)	–	–
Residence	Rural	73	38(52.1)	ref	ref	
	Urban	194	87(44.8)	0.75(0.44--1.28)	2.32(0.73–7.34)	0.152
	Suburban	51	29(56.9)	1.21(0.59--2.49)	4.71(1.44–15.39)	0.010*
Occupation	Gov,t	108	48(44.4)	ref		
	Student	29	16(55.2)	1.54(0.68--3.51)	–	–
	Farmer	66	38(57.6)	1.70(0.91--3.15)	–	–
	Merchant	51	20(39.2)	0.81(0.41--1.59)	–	–
	Housewife	51	29(56.9)	1.65(0.84--3.23)	–	–
	Others	13	3(23.1)	0.38(0.10--1.44)	–	–
Tea consumption	Never	106	59(55.7)	ref	ref	
	One cup/day	96	57(59.4)	1.16(0.67--2.04)	0.99(0.47--2.07)	0.975
	>one cup/day	116	38(32.8)	0.39(0.23--0.67)	0.34(0.17--0.70)	0.003*
Raw fruit/ Vegetable consumption	Never	78	49(62.8)	ref	ref	
	Eat once/day	53	22(41.5)	0.42(0.21--0.86)	0.31(0.13--0.77)	0.011*
	Eat once/week	187	83(44.4)	0.47(0.28--0.81)	0.48(0.24--0.98)	0.042*
Recent drug consumption	Never	187	67(35.8)	ref	ref	
	Aspirin/NSAID	87	61(70.1)	4.20(2.43--7.27)	5.29(2.82--9.93)	<0.001*
	Other drugs	44	26/59.1	2.59(1.32--5.06)	3.12(1.46--6.69)	0.003*
Blood group	Group A	82	37/45.1	ref		
	Group B	89	35/39.3	0.79(0.43--1.45)	–	–
	Group AB	21	12/57.1	1.62(0.62--4.27)	–	–
	Group O	126	70/55.6	1.52(0.87--2.66)	–	–
*H. pylori* infection	No	219	87/39.7	ref	ref	
	Yes	99	67/67.7	3.18(1.93--5.24)	4.33(2.41--7.76)	<0.001*

### Correlation of *H. pylori* infection to self-reported dyspepsia symptoms

Univariate analysis showed that *H. pylori* infection is significantly correlated with upper GI complaints such as PPF, ES, Epigastric burning/pain, nausea as well as their overlaps (Table 6).

**Table 6:** Regression analysis on comparison of self-reported upper gastrointestinal symptoms between *H.pylori* (+) (*n* = 99) and *H.pylori* (−) (*n* = 219) patients, Dessie Referral Hospital, 2015

Self-reported Upper gastrointestinal symptoms		HP(+) = n (%)	HP(−) = n (%)	COR(CI)	p-value	AOR(CI)	p-value
PPF	Yes	41(41.5)	42(19.2)	2.98(1.76–5.02)	<0.001	–	–
	No	58(48.5)	177(80.8)	ref			
ES	Yes	39(39.4)	33(15.1)	3.66(2.12–6.33)	<0.001	2.68(1.38–5.20)	0.004*
	No	60(60.6)	186(84.9)	ref		ref	
Epigastric burning/pain	Yes	58(58.6)	76(34.7)	2.66(1.64–4.33)	<0.001	1.92(1.07--3.43)	0.028*
	No	41(41.4)	143(65.3)	ref		ref	
PPF & ES	Yes	34(34.3)	28(12.8)	3.57(2.01–6.33)	<0.001	–	–
	No	65(65.7)	191(87.2)	ref			
UD & HB	Yes	40(40.4)	38(17.4)	3.23(1.90–5.50)	<0.001	–	–
	No	59(59.6)	181(82.6)	ref			
Belching	Yes	40(40.4)	40(18.3)	3.47(1.05--12.57)	0.050	4.70 (1.54–14.4)	0.007*
	No	59(59.6)	179(81.7)	ref		ref	
vomiting	Yes	9(9.1)	9(4.1)	2.33(0.90–6.07)	0.082	–	–
	No	90(90.9)	210(95.9)	ref			
Nausea	Yes	7(7.1)	4(1.8)	4.09(1.17--14.31)	0.028	3.91(1.03–14.87)	0.045*
	No	92(92.9)	215(98.2)	ref		ref	
UD & bleaching	Yes	33(33.3)	33(15.1)	2.82(1.61–4.93)	<0.001	0.28(0.06–1.01)	0.051
	No	66(66.7)	186(84.9)	ref		ref	
UD, HB & bleaching	Yes	25(25.3)	18(8.2)	3.77(1.95–7.31)	<0.001	–	–
	No	74(74.7)	201(91.8)	ref			

Key: PPF = postprandial fullness, ES = early satiation, AOR = adjusted oddis ratio, COR = crude oddis ratio, HP = *H. pylori*, HB = heart burn, CI = confidence interval, UD = uninvestigated dyspepsia

Constructing a multivariate logistic regression model in which the *H.pylori* (+)/(−) variable taken as the dependent variable, and upper GI symptoms which were found to be significantly correlated in the univariate analysis as independent variables showed that *H. pylori* infection rate were significantly correlated with ES [AOR = 2.68, 95% CI 1.38–5.20], epigastric burning/pain [AOR = 1.92, 95% CI 1.07–3.43], belching [AOR = 4.7, 95% CI 1.54–14.40] and nausea [AOR = 3.91,95%CI 1.03–14.87] (Table 6).

## Discussion

In the present study, 63.5% of study participants had at least one GI symptom. Park et al. in Korea reported that among new consecutive patients presented with chronic GI symptoms 73.9% had upper gastroduodenal symptoms [4]. This variation could be due to differences in socioeconomic and life style factors contributing for gastrointestinal disorders.

In our study, using Rome III diagnostic criteria, the prevalence of UD was 48.8% which is higher than study conducted in African countries such as Nigeria (37.5%) [29] and Uganda (38.9%) [30], but the diagnostic criteria (Rome II) are quite different from current study. Our result is also higher than study conducted in Asian countries such as Korea (13.4%) [11], China (5.67%) [31], Taiwan (32.4%) [32], west part of Iran (41.2%) [12]; and European countries like UK (38%) [22] and Italy (15.1%), where Rome II criteria is employed as main diagnostic criteria [20]. However, our result is lower than another study conducted in Nigeria with 69.1% prevalence of UD among upper gastrointestinal symptomatic patients [33].

The observed variable proportion of UD may be partly explained with variation in geographical location, use of variable definitions and criteria for diagnosis of dyspepsia, study design and population. For instance, some studies employed Rome III criteria for diagnosis of dyspepsia [31–33] which is comparable to our work, but the design of most mentioned studies, whether employed Rome II or III criteria, [12, 20, 22, 29–32] are population based surveys involving asymptomatic patients. However, the high proportion of UD in our and Nigerian [33] study is due to being hospital based study where clinically suspected patients involved. Moreover, in our and other African studies [29, 30, 33] the prevalence of UD is higher as compared to studies conducted in Asia. This might be delayed presentation of patients to health care provider in African countries and the result of variation in dietary factors, socio-cultural and psychological issues, rate of gastrointestinal infection mainly caused by *H. pylori.* As a result eastern life styles may be a factor related to the lower prevalence dyspepsia.

The most common symptoms associated with UD was found to be epigastric pain/burning (42.1%), PPF (26.1%), ES (22.6%) and belching (25.2%) (Table 2). Similarly, among Ugandan patients presenting with upper GI symptoms the most common presenting complaint was epigastric pain (51.6%) [34]. Our result is also in line with study in Iran as epigastric pain or burning (58.3%) being dominant complaint of dyspeptic patients followed by PPF (37.3%) and ES (18.6%) [35]. Belching disorders (30.7%) was the dominant symptoms next to FD (46%) among functional gastroduodenal disorders in Korean patients with chronic GI complaints [4].

Correlation of *H. pylori* infection with each upper GI symptoms is shown in Table 6. There was no correlation between PPF, combination of UD and heart burn, and vomiting with *H. pylori* infection. However, significant correlation was found between ES, epigastric pain/burning, belching, and nausea with *H. pylori* infection. The proportion of belching is more among *H. pylori* infected patients as compared to *H. pylori* free patients. i.e., presentation of belching is 4.7 times more likely to be associated with *H.pylori* infection as compared to *H. pylori* free patients. Our result is in agreement to report of Shokrzadeh et al. in Iran where the prevalence of belching was significantly higher among *H. pylori* infected patients with no correlation of vomiting [36]. This significant likelihood association *H. pylori* infection to belching could be explained as *H. pylori* is known to produces urease which helps to metabolizes urea into ammonia and carbon dioxide resulting in belching and bloating**.** However, our result is found contrary to his report where no difference was observed in the prevalence of epigastric abdominal pain, nausea, and ES between *H. pylori*-positive and *H. pylori*-negative patients [36]. This may be due to variation in laboratory tests for diagnosis of *H. pylori* infection (stool antigen detection in our case where as histological examination in Iranian study), population variation and lifestyle factors.

The association of *H. pylori* infection with UD has been reported with inconsistent findings in several studies. In our study, however, *H. pylori* infection was found to be significantly associated with UD (AOR = 4.33, 95% CI 2.41–7.76, *p* < 0.001) which is similar to study conducted in Taiwan [32], Kenya [37] and Ethiopia [23]. A large population based study in Denmark also showed that *H. pylori* infection is a significant risk factor for dyspepsia although of less importance than NSAIDs use, unemployment and heavy smoking [38]. However, other authors indicated no association between *H. pylori* infection and dyspepsia [18–20, 39] as most *H. pylori* infected individuals didn’t show dyspepsia [40–43].

Even though some surveys have indicated association of dyspepsia with alcohol consumption [44, 45], our result showed no significant association between coffee and alcohol consumption with UD which is in agreement with studies conducted elsewhere in the world reporting no association with dyspepsia [17, 19, 20, 39, 46, 47]. This discordance could be due to difference in type and amount of alcohol consumed as well as sociocultural practices of study population. Moreover, in our study in addition to small sample size we didn’t determine the amount and type of consumed alcohol, as well as the type of coffee consumed so that further population-based study is needed to elucidate their association with dyspepsia.

Our result also showed that NSAIDs use is statistically associated with UD (AOR = 5.29, 95% CI 2.82–9.93, *p* < 0.001) which is in agreement with British and American populations based studies where NSAIDs use have been identified as an important independent risk factor for UD [22, 32, 46]. However, population based study in Italy and Sweden showed no association between use of NSAIDs/aspirin or corticosteroids and UD [20, 39]. Sex and age matched case control study conducted in Nigeria showed that NSAIDs user for a minimum of one week prior to 30 days before enrolment to the study did not show any association with UD [19].

Our study also showed that consumption of two or more cup of tea a day (AOR = 0.34, 95%CI 0.17–0.70, *p* = 0.003) and consumption of raw fruits and/or vegetables at least once a day (AOR = 0.31, 95% CI 0.13–0.77, *p* = 0.011) was protective against UD which is in agreement with reports of Khademolhosseini et al. in Iran, where inverse relationship was observed between dyspepsia and fruits as well as vegetables consumption [47]. However, Seyedmirzaei et al. in Iran, by controlling possible confounding factors, showed no protective effect of high fruit and vegetable consumption, and current tea-coffee consumption [35]. Hospital-based study in Taiwan also showed no significant association between consumption of tea, vegetable and UD, but consumption of coffee was protective against UD [32]. Contrary to above findings, Solomon et al. in Nigeria indicated significant association of high amount of tea consumption and UD [19]. This may depend on the type of tea, fruit and vegetables as well as type of biological molecules that can be found within it, which is not investigated in our study and others too. Thus, future well designed study is warranted to assess whether different kinds of tea as well as fruit and vegetables have different biological effects in the pathogenesis of dyspepsia.

The strength of our study is that we have included new patients by using randomized recruitment strategy to represent all patients with symptoms of GI disorders. However, the cross-sectional nature of this study has a limitation to show cause and effect associations between UD and associated factors, and the result cannot be representative of the general population. This study is a hospital based study involving subjects with symptoms who have had many underline disease conditions as a confounding factors that could not fully controlled so that it might have impact on the statistical correlations between dyspepsia and other variables of interest.

## Conclusions

In conclusion, this is the first hospital-based study in Ethiopia for assessing UD among patients with FGIDs using Rome III criteria. The prevalence of UD in the study population was 48.4%, with epigastric pain/burning being the most prevalent symptom (42.1%). The observed high proportion of UD alerts health care providers and program planners about the disease, and suggesting a critical management issue of dyspepsia in the country. *H. pylori* infection is significantly associated with UD and habit of aspirin/NSAIDs consumption. However, consumption of raw fruit and/or vegetable at least once a day and more than one cup of tea a day showed inverse association with UD which needs further well designed study to ascertain the observed association. Upper GI symptoms such as early satiation, epigastric burning/pain, belching, and nausea showed strong likelihood association with *H. pylori* infection.

## Abbreviations

ES: early satiation
FD: functional dyspepsia
FGIDs: functional gastrointestinal disorders
GI: gastrointestinal
NSAIDS: nonsteroidal anti-inflammatory drugs
PPF: post prandial fullness
UD: uninvestigated dyspepsia
